# The effects of the voglibose on non-alcoholic fatty liver disease in mice model

**DOI:** 10.1038/s41598-022-15550-7

**Published:** 2022-08-10

**Authors:** Jaehyun Bae, Ji Young Lee, Eugene Shin, Minyoung Lee, Yong-ho Lee, Byung-Wan Lee, Eun Seok Kang, Bong-Soo Cha

**Affiliations:** 1 Department of Medicine, Graduate School, Yonsei University College of Medicine, Seoul, South Korea; 2 Division of Endocrinology and Metabolism, Department of Internal Medicine, International St. Mary’s Hospital, Catholic Kwandong University College of Medicine, Incheon, South Korea; 3 Institute of Endocrine Research, Yonsei University College of Medicine, Seoul, South Korea; 4 Division of Endocrinology and Metabolism, Department of Internal Medicine, Yonsei University College of Medicine, 50 Yonsei-ro, Seodaemun-gu, Seoul, 03722 South Korea

**Keywords:** Endocrinology, Gastroenterology

## Abstract

The α-glucosidase inhibitor (α-GI) delays the intestinal absorption of glucose, which reduces postprandial hepatic glucose intake. This mechanism is considered to be effective in treating non-alcoholic fatty liver disease (NAFLD). Here, we investigated the effect of voglibose, an α-glucosidase inhibitor, on high-fat, high-fructose (HFHFr) diet-induced NAFLD models. Seven-week-old male C57BL/6J mice were randomly placed in a chow diet group or an HFHFr diet group. After 10 weeks, mice in the HFHFr group were randomly assigned to one of three groups: HFHFr diet with vehicle, HFHFr with voglibose, or HFHFr with pioglitazone. Each diet and treatment was continued for 10 weeks. The HFHFr diet induced severe NAFLD in terms of steatosis, hepatitis, and fibrosis. Administration of voglibose improved all aspects of NAFLD, comparable to those of pioglitazone, a positive control. In voglibose-treated mice, gene expressions of hepatic lipogenesis markers were significantly downregulated. In the in vitro experiment, reducing the influx of glucose into hepatocytes significantly reduced steatosis and de novo lipogenesis even in the presence of sufficient fructose and fat, demonstrating that the mechanism of voglibose could be effective in treating HFHFr diet-induced NAFLD. These results indicate that voglibose improves HFHFr diet-induced NAFLD by suppressing hepatic de novo lipogenesis.

## Introduction

Non-alcoholic fatty liver disease (NAFLD) is a chronic metabolic disease in which abnormal amounts of lipids accumulate in the liver and its prevalence is increasing worldwide. Approximately 25% of the global population is affected by this disease^1^. Its increasing prevalence is considered to be associated with unhealthy lifestyles, particularly unhealthy diets^2^. Increased intake of glucose, fructose, and saturated fat increases hepatic de novo lipogenesis (DNL) as well as systemic insulin resistance and low-grade inflammation, both of which promote hepatitis and hepatic steatosis. Prolonged hepatitis can become cirrhosis or hepatocellular carcinoma^3,4^.

To improve or treat NAFLD, weight loss through lifestyle modification is preferentially recommended^5,6^, and in severely obese cases, bariatric surgery can also be considered^7^. However, there is still a lack of effective pharmacological NAFLD treatments. Most treatment guidelines recommend the use of vitamin E, an antioxidant, or the antidiabetic agent pioglitazone for patients with type 2 diabetes^2,7–10^, but they were not widely used due to limited efficacy or side effects. Recently, therapeutic or preventive effects of glucagon-like peptide-1 (GLP-1) agonists or sodium glucose cotransporter-2 (SGLT-2) inhibitors for NAFLD have been actively studied^11–14^, but they have not yet been recognized as a formal option for NAFLD treatment.

High carbohydrate intake is an important factor in NAFLD pathogenesis^2,15^, so α-glucosidase inhibitors (α-GIs), which delay the absorption of glucose in the lumen of the small intestine, may be used to treat NAFLD. By slowing glucose absorption in the intestine, α-GIs suppress surges of postprandial blood glucose levels and hepatic glucose intake^16^. Some studies have evaluated the preventive effects of α-GIs for NAFLD in animal models^17–19^. In addition, a clinical, single-arm study showed that α-GI has a therapeutic effect in histologically-confirmed NAFLD patients with diabetes^20^.

However, there is a lack of studies on the effects of α-GIs in nondiabetic NAFLD models, especially with regard to their therapeutic effects. Furthermore, no study has yet examined the role of α-GIs in NAFLD models induced by high-fat, high-fructose (HFHFr) diets, which is a major factor in NAFLD pathogenesis.

Therefore, in this study, we examined the therapeutic effect of the α-GI voglibose on the HFHFr diet-induced nondiabetic NAFLD mouse model. Through 10 weeks of HFHFr diet, NAFLD was induced in mice. Thereafter, the mice were randomly subdivided into three groups: HFHFr diet with vehicle (HFHFr group), HFHFr diet with voglibose (HFHFr-V group), and HFHFr diet with pioglitazone group (HFHFr-P group). Each group received the vehicle or the agent for 10 weeks, and after the end of the experiment, we evaluated the effect of the agent on NAFLD.

## Results

### Biochemical characteristics

During the 10-week NAFLD-induction period, the average body weight of the HFHFr group increased significantly more than that of the chow diet group, but they had similar random blood glucose levels (Fig. S1). During the 10-week treatment period, the average body weight of the HFHFr-V group increased less than those of the HFHFr and HFHFr-P groups. All four groups had similar random blood glucose levels.

At the end of the 20 weeks of experimentation, the HFHFr group’s average body weight, liver weight, and liver enzyme levels were elevated, indicating that they had NAFLD (Table 1), whereas diabetes was not induced according to the blood glucose levels and the results of oral glucose tolerance test (OGTT, data not shown). Mice which received voglibose showed a trend of lower body weight and liver weight. In addition, the HFHFr-V group showed improved liver enzymes levels. Pioglitazone treatment lowered liver enzyme levels, but not to a statistically significant degree. There were no statistically significant differences in fasting blood glucose levels and insulin resistance as assessed by homeostasis model assessment method (HOMA-IR)^21^, between the three HFHFr-fed groups.

**Table 1:** Body weight and biochemical measurements at 20th week.

	Chow	HFHFr	HFHFr-V	HFHFr-P
Body weight (g)	27.0 ± 0.9	38.2 ± 3.4^#^	33.0 ± 2.8^#†^	35.0 ± 2.0^#^
Liver weight (g)	1.1 ± 0.1	3.0 ± 0.7^#^	1.8 ± 0.4^#†^	2.6 ± 0.4^#^*
Liver to body weight (%)	4.2 ± 0.1	7.9 ± 1.2^#^	5.3 ± 0.7^#†^	7.4 ± 0.8^#^*
AST (IU/L)	82.6 ± 17.4	463.8 ± 250.8^#^	154.2 ± 66.1^†^	288.4 ± 96.8
ALT (IU/L)	26.0 ± 6.4	487.0 ± 303.1^#^	114.6 ± 88.8^†^	255.6 ± 99.7
Fasting glucose (mg/dL)	133.6 ± 27.8	235.8 ± 118.4	177.0 ± 75.5	184.1 ± 31.8
Fasting insulin (ng/dL)	0.34 ± 0.02	0.25 ± 0.004^#^	0.25 ± 0.01^#^	0.26 ± 0.004^#^
HOMA-IR	2.8 ± 0.6	3.7 ± 1.9	2.4 ± 0.9	2.9 ± 0.5

HFHFr, high-fat, high-fructose diet group; HFHFr-V, HFHFr diet with voglibose group; HFHFr-P, HFHFr diet with pioglitazone group; AST, aspartate aminotransferase; ALT, alanine aminotransferase; HOMA-IR, homeostasis model assessment of insulin resistance.

^#^*P* < 0.05 vs. chow, ^†^*P* < 0.05 vs. HFHFr, *P < 0.05 vs. HFHFr-V.

### The effect of voglibose on hepatic steatosis, inflammation, and fibrosis

**Figure 1:**
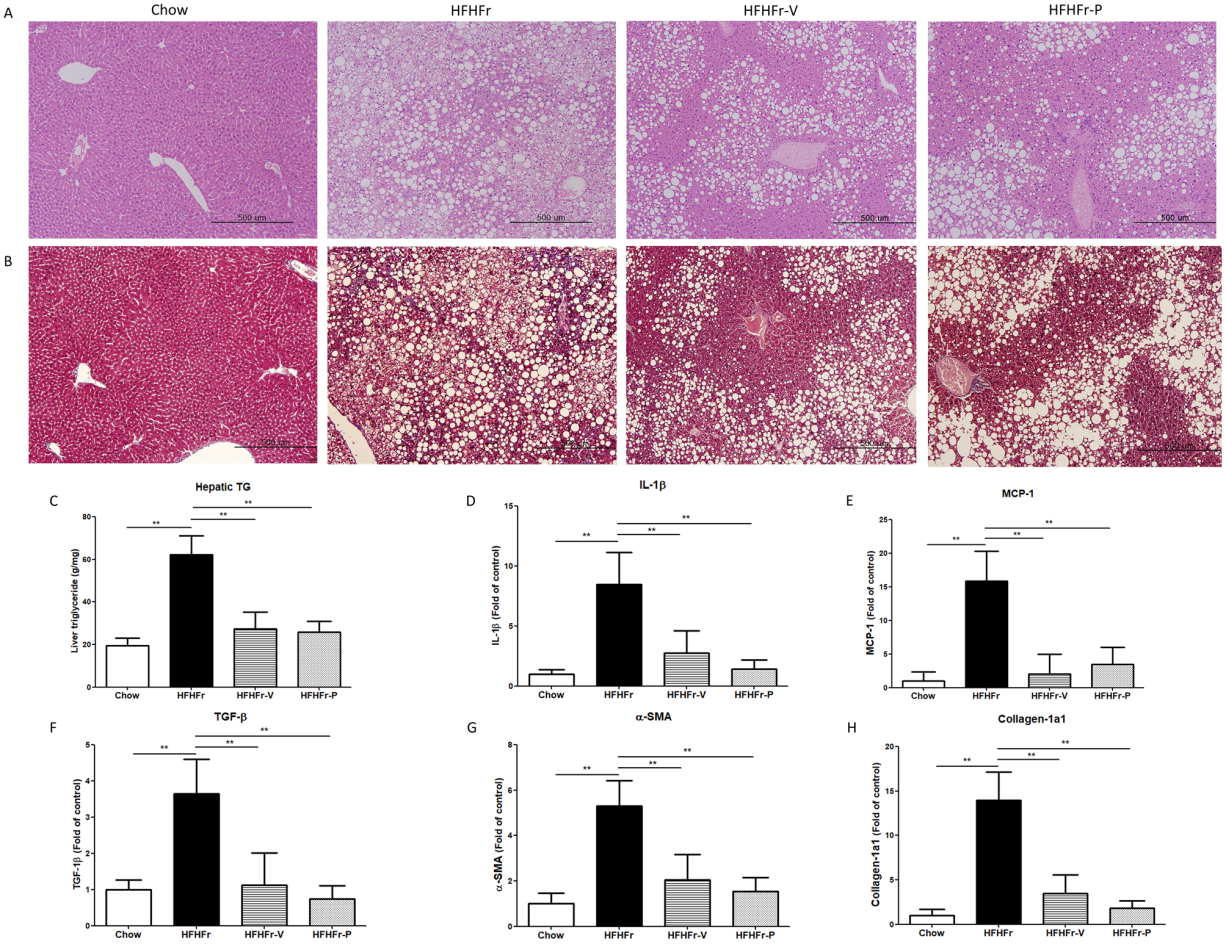
Effects of voglibose on non-alcoholic fatty liver disease. (**A**) Histopathological images of the hematoxylin and eosin stained liver tissue, and (**B**) Masson’s trichrome stained liver tissue. (**C**) Hepatic triglyceride (TG) level, mRNA expression levels of (**D**) interleukin-1β (IL-1β), (**E**) monocyte chemoattractant protein-1 (MCP-1), (**F**) transforming growth factor-β (TGF-β), (**G**) α-smooth muscle actin (α-SMA), and (H) collagen type 1α1 chain (collagen-1α1). *p < 0.05; **p < 0.01; HFHFr, high-fat, high-fructose diet group; HFHFr-V, HFHFr diet with voglibose group; HFHFr-P, HFHFr diet with pioglitazone group.

Histological assessment for hepatic steatosis was performed on the hematoxylin and eosin-stained liver tissue sections (Fig. 1). A triglyceride (TG) quantification kit was used to quantify the degree of steatosis. We measured the hepatic messenger ribonucleic acid (mRNA) expression of the inflammatory and fibrotic markers, interleukin-1 beta (IL-1β), monocyte chemoattractant protein-1 (MCP-1), transforming growth factor-β (TGF-β), α-smooth muscle actin (α-SMA), collagen type 1α1 chain (Collagen-1a1). The results of these analyses showed that the HFHFr diet induced severe NAFLD in mice as reflected by steatosis, hepatitis, and even fibrosis.

Administration of voglibose improved all aspects of HFHFr diet-induced NAFLD. Quantitative analysis showed that it reduced hepatic TG levels, in line with the histological findings. It also decreased the mRNA expression of inflammatory and fibrotic markers, indicating that it reduced inflammation and fibrosis. These effects were similar to those of pioglitazone, a positive comparator group. In an additional experiment measuring the protein levels of the inflammatory markers, the same trend was obtained (Fig. S2).

### The effect of voglibose on hepatic DNL

**Figure 2:**
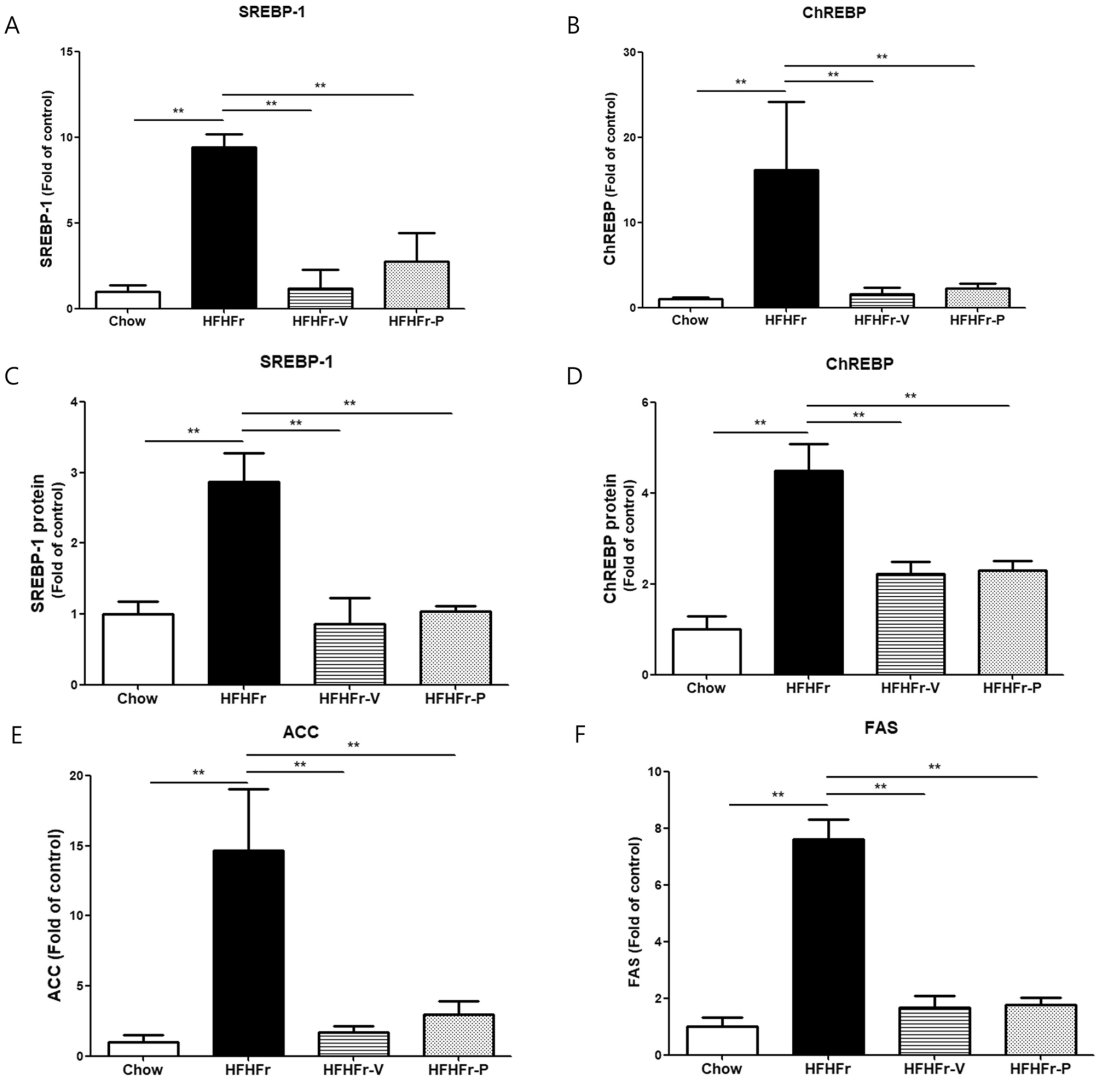
Effects of voglibose on hepatic de novo lipogenesis. mRNA expression levels of (**A**) sterol regulatory element-binding transcription factor-1 (SREBP-1), (**B**) carbohydrate response element-binding protein (ChREBP), protein levels of (**C**) SREBP-1, (**D**) ChREBP, and (**E**) the representative blots image, mRNA expression levels of (**F**) acetyl-CoA carboxylase (ACC) and (**G**) fatty acid synthase (FAS). *p < 0.05; **p < 0.01; HFHFr, high-fat, high-fructose diet group; HFHFr-V, HFHFr diet with voglibose group; HFHFr-P, HFHFr diet with pioglitazone group.

Voglibose reduces glucose absorption by inhibiting α-glucosidase activity in the intestinal lumen, reducing the hepatic inflow of glucose, which is an important material for DNL in the liver. To evaluate whether voglibose treatment reduced hepatic DNL, we measured the mRNA expression and protein levels of sterol regulatory element-binding transcription factor-1 (SREBP-1) and carbohydrate response element-binding protein (ChREBP), key regulators of hepatic DNL^22^, and the mRNA expression of the hepatic lipogenic enzymes acetyl-CoA carboxylase (ACC) and fatty acid synthase (FAS) (Fig. 2 and Fig. S3). The results showed that voglibose strongly reduced the expression of the two major transcriptional regulators of DNL, SREBP1 and ChREBP, and the expression of downstream lipogenic enzymes, ACC and FAS.

In addition, voglibose also reduced the mRNA expression of hepatic gluconeogenesis markers such as phosphoenolpyruvate carboxykinase (PEPCK) and glucose 6-phosphatase (G6Pase) (Fig. S4). This suggests that voglibose can reduce not only hepatic lipogenesis but also gluconeogenesis.

### The effect of reduced glucose inflow to hepatocytes on hepatic lipogenesis

**Figure 3:**
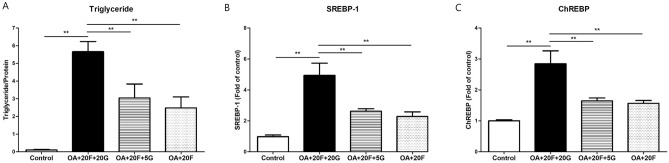
Effects of reduced glucose inflow to hepatocytes on hepatic lipogenesis. (**A**) Hepatic triglyceride level, mRNA expression levels of (**B**) sterol regulatory element-binding transcription factor-1 (SREBP-1) and (C) carbohydrate response element-binding protein (ChREBP). *p < 0.05; **p < 0.01; OA + 20F + 20G, HepG2 treated with 1.0 mM oleic acid (OA), 20 mM fructose and 20 mM glucose for 48 h; OA + 20F + 5G, HepG2 treated with 1.0 mM OA, 20 mM fructose and 5 mM glucose for 48 h; OA + 20F, HepG2 treated with 1.0 mM OA and 20 mM fructose.

In this study, we used the HFHFr diet that had high contents of lipogenic ingredients, such as fat, cholesterol, and fructose. Although there were some portions of other carbohydrates, it was hypothesized that voglibose would have limited influence on hepatic lipogenesis. However, our results showed that it did have therapeutic effects. We hypothesized that this result was likely a product of the fact that reducing glucose inflows to hepatocytes even in diets with high concentrations of lipids and fructose significantly reduced hepatic lipogenesis. Thus, we designed an additional experiment in which HepG2 cells were treated with 1.0 mM Oleic acid (OA) and 20 mM fructose, which mimicked an HFHFr diet, for 48 h with 0, 5, or 20 mM glucose. Hepatocytes treated with OA, fructose, and 5 mM glucose showed significantly lower levels of hepatic TG than those treated with OA, fructose, 20 mM glucose (Fig. 3). Cells treated with 5 mM glucose also showed lower mRNA expression levels of SREBP-1 and ChREBP than those treated with 20 mM glucose. This result indicates that reducing glucose inflow to hepatocytes can limit lipogenesis even in the presence of high concentrations of lipids and fructose.

## Discussion

In this study, we investigated the therapeutic effects of voglibose, an α-GI, on HFHFr-induced NAFLD in nondiabetic mice. Although the HFHFr diet used in this experiment might be unfavorable to show voglibose’s therapeutic effects on NAFLD, treatment with voglibose for 10 weeks significantly improved NAFLD in terms of hepatic steatosis, inflammation, and fibrosis. These effects were similar to those caused by pioglitazone, a drug recommended for NAFLD treatment by various guidelines and reviews^2,3,8^. There have been several previous studies on the preventive effects of the α-GIs for NAFLD, but our study is the first study to show the therapeutic effect of the α-GI for nondiabetic NAFLD model induced by HFHFr diet (Table 2).

**Table 2:** Studies on the effects of α-glucosidase inhibitors in NAFLD animal models.

Studies	Animal	Diet	α-GI	Effects on NAFLD
Bae et al	C57BL/6J mice	High-fat (40%), high-fructose (20%) diet	Voglibose	Less steatosis, inflammation, and fibrosis
Lieber et al.^17^	Sprague–Dawley rats	High-fat (71%) diet	Acarbose	Less steatosis and inflammation
Okada et al.^18^	Sequestosome 1/A170/p62 deficient mice	Chow diet	Acarbose	Less steatosis
Nozaki et al.^19^	C57BL/6J mice	High-fat (57.5%) diet	Acarbose	Less steatosis, inflammation, and fibrosis
Kim et al.^23^	OLETF rats	18% protein rodent diet	Voglibose	Less steatosis

α-GI, α-glucosidase inhibitor; NAFLD, non-alcoholic fatty liver disease; OLETF rats, The Otsuka Long‐Evans Tokushima fatty rats.

Voglibose is poorly absorbed after oral administration causing the majority of the drug to remain in the intestinal lumen after which it is rapidly excreted in stool^24,25^. To date, no voglibose metabolites have been identified. Therefore, we focused on the action of voglibose in the intestinal lumen, inhibition of intestinal glucose absorption. By inhibiting α-glucosidase activity, voglibose delays and thereby reduces the total amount of glucose uptake through enterocytes, which decreases glucose inflow to the liver and reduces the rate at which postprandial blood glucose levels increase. Glucose is a significant part of DNL in the liver^26^, which is considered as a prominent pathophysiological abnormality of NAFLD^27^. We hypothesized that the therapeutic effects of voglibose on NAFLD were because it reduced hepatic DNL. As was hypothesized, voglibose treatment strongly suppressed the expression of the two major transcriptional regulators DNL, SREBP1 and ChREBP, and suppressed the expression of the downstream lipogenic enzymes ACC and FAS. These results reflect the decrease in hepatic DNL, which is the main mechanism by which voglibose affects NAFLD. The results of in vitro experimentation showed how reduced glucose uptake into hepatocytes had a significant effect on DNL even in HFHFr-fed mice, supporting the conclusion that voglibose treatment can have a therapeutic effect on HFHFr-induced NAFLD.

Pioglitazone is an antidiabetic agent that improves insulin sensitivity by activating peroxisome proliferator-activated receptor γ (PPARγ)^28^. In addition to improving insulin sensitivity, pioglitazone has various systemic effects, such as anti-inflammatory, vasoprotective, and autophagy-inducing effects^29–31^. As mentioned above, pioglitazone is the only antidiabetic agent generally recommended by NAFLD management guidelines. Interestingly, it has been reported that pioglitazone improves NAFLD even in the patients without diabetes^32,33^. Although it is not clear whether PPARγ activation is more prominent in lipids in adipose tissue than those in the liver^34–36^, most clinical studies have reported that pioglitazone reduced hepatic steatosis^9,32,33^. In addition, those studies also reported that pioglitazone improved hepatic inflammation. In this study, pioglitazone treatment improved steatosis, inflammation, and fibrosis in nondiabetic NAFLD mice.

Relatively novel antidiabetic agents such as GLP-1 agonists or SGLT-2 inhibitors, have gained attention as candidate NAFLD treatments^2,7^. These agents also cause weight loss and have cardioprotective effects and so could be particularly effective for treating NAFLD in patients with diabetes^11–14,37,38^. In this study, the α-GI, a relatively old-fashioned antidiabetic drug, was shown to have a comparable effect as pioglitazone in treating NAFLD. Although this study was an animal study, its results are noteworthy because they showed that voglibose had a marked therapeutic effect in the nondiabetic NAFLD model induced by the HFHFr diet, which resembles contemporary unhealthy diets.

Our study had two limitations. The first limitation was the method of drug administration. It would be ideal to administer voglibose before every meal, considering its action mechanism. Therefore, in mice experiments, voglibose could be mixed with their food. However, this would change the food’s properties and a considerable amount of the drug would be lost, making it hard to ensure that mice were receiving the appropriate dosage. Thus, we decided to administer the drug via oral gavage once daily. The second limitation was that we did not investigate the therapeutic mechanisms of voglibose other than in how it reduces glucose inflows to the liver, such as how it affects gut microbiota. In fact, previous studies have reported that α-GIs may affect the composition of the gut microbiota^39,40^, and evidence is accumulating that gut microbiota play a role in NAFLD^41,42^. Future studies should investigate how α-GIs influence gut microbiota and how this relationship could be used to treat NAFLD. In addition, clinical studies should be conducted that evaluate the therapeutic effects of α-GIs on NAFLD patients with or without diabetes.

In conclusion, administration of voglibose improved NAFLD in terms of hepatic steatosis, inflammation, and fibrosis in HFHFr-fed nondiabetic mice. Voglibose reduced glucose inflows to the liver, which suppressed hepatic DNL even in spite of the mice’s HFHFr diet. This result suggests that α-GIs could be effective at treating NAFLD in humans, even those whose diets are high in fructose and fat.

## Methods

### Animal experiments

Seven-week-old male C57BL/6J mice were purchased from the Jackson Laboratory (Bar Harbor, ME, USA). After 1-week acclimatization period, the mice were randomly divided into two groups. One group received a chow diet (N = 6) and the other received an HFHFr diet (N = 30). After 10 weeks on their respective diets, mice in the HFHFr group were randomly subdivided into three groups: HFHFr group (N = 10), HFHFr-V group (N = 10), and HFHFr-P group (N = 10) groups. Mice in the chow diet group maintained the same diet and received daily vehicle administration. Each treatment was administered daily by oral gavage for 10 weeks. Voglibose and pioglitazone doses were 1.0 mg/kg/day and 10 mg/kg/day, respectively, given human doses and the results of previous experiments^43–46^. The mice were maintained at a temperature of 23 °C ± 2 °C and a humidity level of 60% ± 10% under a 12-h light/dark cycle.

Regular chow diet (PicoLab Rodent Diet 20 [5053]) contained 23.6% protein, 64.5% carbohydrate, and 11.9% fat (% of total kcal), and the HFHFr diet (Catalog number D17010102; Research Diets, New Brunswick, NJ, USA) contained 20% protein, 40% high fat (of these 22.7% trans-fat), 20% fructose (% of total kcal), and high cholesterol (2% by weight). The HFHFr diet was selected to induce NAFLD in mice because its composition was similar to that of the Amylin liver NASH diet which has been shown to induce significant NAFLD in mice^47,48^. Diets were provided daily to eat ad libitum.

At the end of the 20-week experimental period, consisting of 10 weeks of NAFLD-induction followed by 10 weeks of treatment, the mice were anesthetized and sacrificed 24 h after the final administration. All animal procedures were approved by the Animal Care and Use Committee at the Yonsei University College of Medicine (approval number: 2018–0180) and all experiments were performed in accordance with relevant regulations and guidelines including the ARRIVE guideline.

### Biochemical measurement and histological analysis

Random blood glucose concentrations were assessed weekly around 2 pm by tail vein sampling. Body weight was also measured weekly over the entire treatment period. OGTT was performed at week 20 of the experiment. At sacrifice, blood was collected via heart puncture and liver tissues were harvested. Serum levels of aspartate aminotransferase, alanine aminotransferase, glucose, and insulin were measured. Insulin resistance was assessed using HOMA-IR. Liver specimens were flash-frozen in liquid nitrogen and maintained at -80 °C until analysis. Hepatic TG levels were determined using a K622TG quantification kit (Biovision) according to the manufacturer’s instructions.

Liver sections were histologically assessed using an Olympus BX40 light microscope (Olympus Optical Co., Ltd., Tokyo, Japan) and 5 mm × 5 mm sections were fixed in 4% paraformaldehyde for 48 h then embedded in paraffin. A microtome (Reichert Scientific Instruments, Buffalo, NY, USA) was used to prepare 4 μm-thick tissue sections that were placed on glass slides. Paraffin-embedded liver specimens were stained with hematoxylin and eosin or Masson’s trichrome protocol.

### Ribonucleic acid isolation and real-time polymerase chain reaction analysis

Total RNA was extracted using a Hybrid-R RNA purification kit (GeneAll Biotechnology, Seoul, South Korea) according to the manufacturer’s instructions. Complementary deoxyribonucleic acid (cDNA) was synthesized using high-capacity cDNA reverse transcription kit (Applied Biosystems, 4,368,814) and 2.5 μM random primers. Quantitative real-time polymerase chain reactions (PCR) were performed in 10 μl reactions containing 1.0 μl cDNA, 5 pmol of each oligonucleotide primer, and 5.0 μL of Power SYBR Green PCR Master Mix (Applied Biosystems, 4367659). Quantitative real-time PCR was performed using the 2^−ΔΔCt^ method and a StepOnePlus Real-Time PCR System (Applied Biosystems, Foster City, CA, USA) in a 96-well plate. The expression of target genes was normalized to that of the reference gene, 18S ribosomal RNA. The expressions of 18S, *Il-1β*, *Mcp-1*, *Tgf-β*, *α-Sma*, *Collagen-1a1*, *Srebp-1*, *Chrebp*, *Acc*, *Fas, PEPCK, and G6pase* were assessed using specific primers, of which sequences are listed in Supplementary Table 1.

### Western blot analysis

Protein was extracted from liver tissue by homogenization in lysis buffer (T-PER Reagent; Thermo Scientific, Rockford, IL. catalogue number 78510) supplemented with protease and phosphatase inhibitors (Thermo Scientific, Rockford, IL. catalogue number 78441), according to manufacturer’s instructions. Samples were centrifuged to pellet tissue debris. For each assay, 20 μg of protein was used.

Protein concentrations were determined using clear supernatants by Bradford reagent (Sigma Aldrich) and bovine serum albumin as a control. Then 20 μg of total protein was electrophoresed in sodium dodecyl sulfate polyacrylamide gel electrophoresis 10% gradient gel. Proteins were transferred to polyvinylidene fluoride membranes (Millipore). Samples were immunoblotted overnight with the indicated primary antibodies at a dilution of approximately 1:1,000 and then conjugated with secondary antibodies using horseradish peroxidase at a 1:5,000 dilution. The SuperSignal West Pico Plus Kit (Thermo Scientific) and specific antibodies against GAPDH (catalogue number 32233, Santa Cruz), IL-1β (catalogue number AB1413-I, Sigma-Aldrich), MCP-1 (catalogue number NBP2-22115, Novusvio), SREBP-1 (PA1-46142, Thermo Fisher Scientific) and ChREBP (NB400-135, Novus Biologicals) were used for detection.

### Cell culturing and treatment

The human hepatoma HepG2 cells (American Type Culture Collection, Manassas, VA, USA) were cultured in 5.5 mM low glucose Dulbecco’s modified Eagle’s medium (SH30021.01, HyClone Laboratories, Logan, UT, USA) supplemented with 10% fetal bovine serum, 1% penicillin, and 1% streptomycin in a humidified 5% CO_2_ incubator at 37 °C for 3 days to achieve 70% confluence before treatment. HepG2 cells were treated with OA (Sigma-Aldrich, 1.0 mM) and fructose (20 mM) for 48 h with different concentrations of glucose, 5 mM or 20 mM. After treatment, hepatic TG quantification and quantitative real-time PCR for Srebp-1 and Chrebp were conducted.

### Statistical analysis

Data are expressed as mean ± standard deviation (SD). Analysis of variance followed by Bonferroni’s post hoc test was used to compare variables between the groups. *P*-values < 0.05 were considered to be statistically significant. All statistical analyses were performed using SPSS version 21.0 for Windows (IBM Corp., Armonk, NY, USA).

## Supplementary Information

Supplementary Information 1.

Supplementary Information 2.

## Data Availability

The data that support the findings of this study are available from the corresponding author upon reasonable request.
