# Supplementary material for: The effects of the voglibose on non-alcoholic fatty liver disease in mice model
Source: Sci Rep. 2022 Aug 10;12:13595. doi: 10.1038/s41598-022-15550-7 (PMC9365779; doi:10.1038/s41598-022-15550-7)
Supplement: Supplementary file 1 — Supplementary Information 1. [file 41598_2022_15550_MOESM1_ESM.docx]

**Figure S1. Changes in body weight and random blood glucose levels during the experiment.**


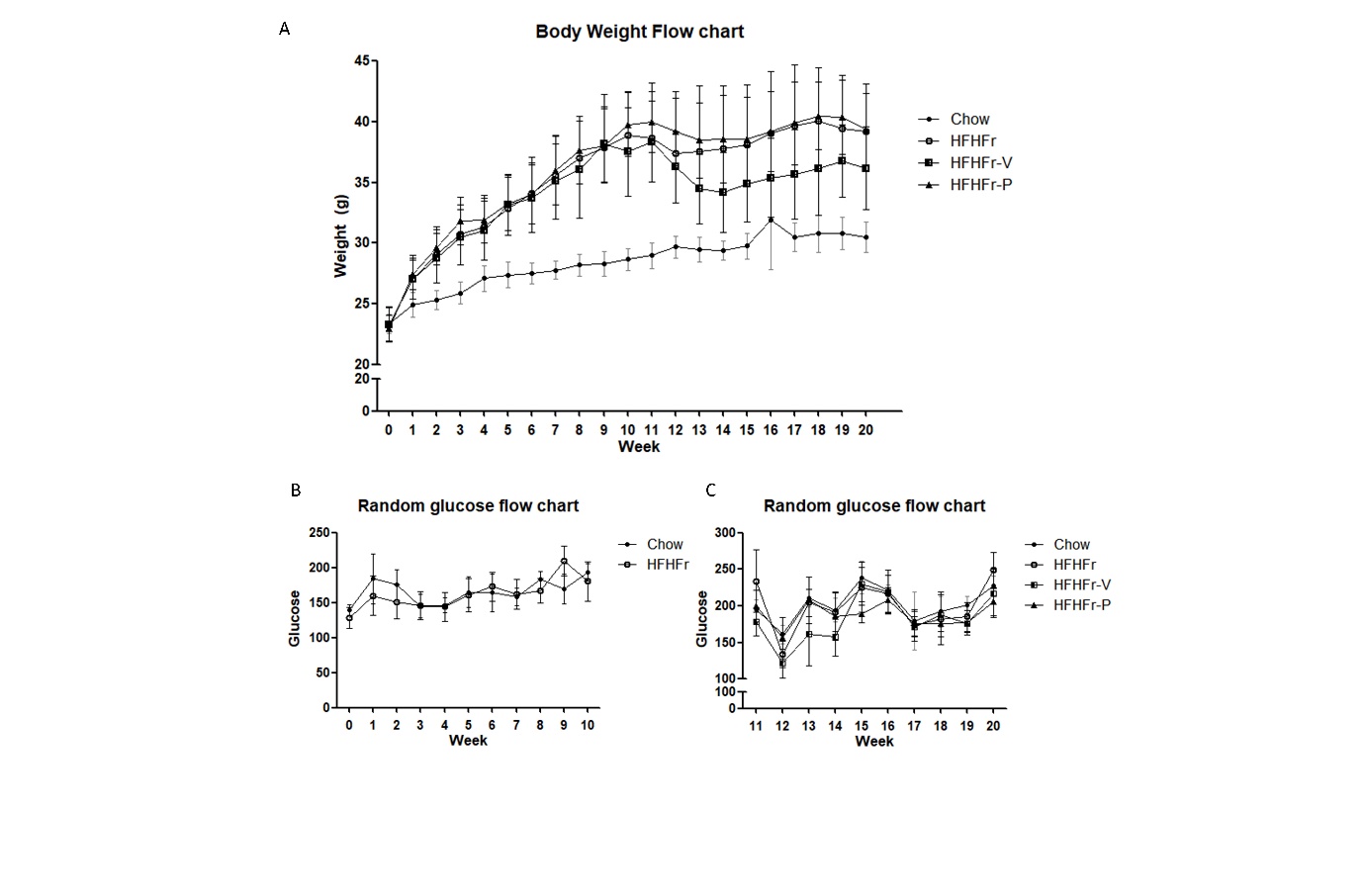


(A) Body weight flow chart during the whole experimental period, (B) random blood glucose flow chart during the first 10 weeks, and (C) random blood glucose flow chart during the latter 10 weeks

HFHFr, high-fat, high-fructose diet group; HFHFr-V, HFHFr diet with voglibose group; HFHFr-P, HFHFr diet with pioglitazone group

**Figure S2. Effects of voglibose on hepatitis**

**
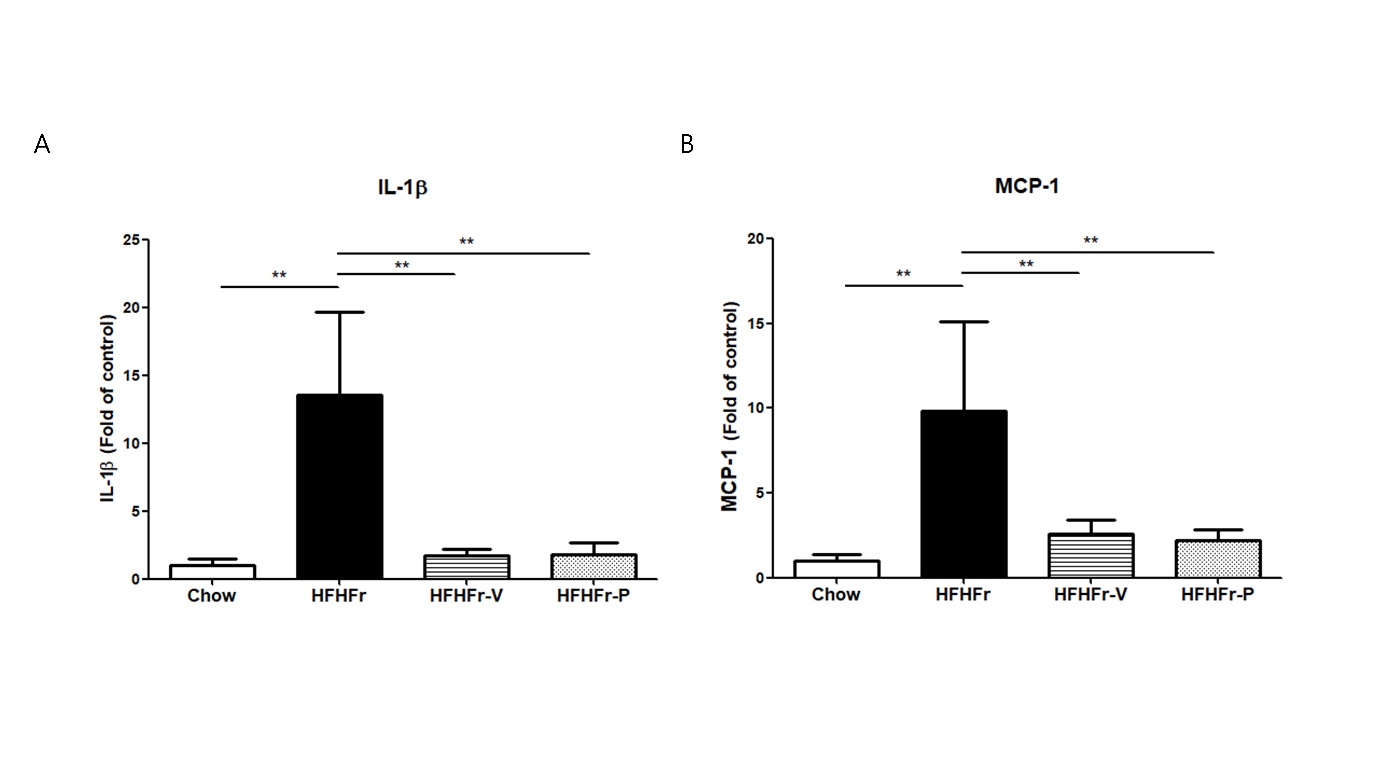
**

Protein levels of (A) interleukin-1β (IL-1β), and (B) monocyte chemoattractant protein-1 (MCP-1)

*p<0.05; **p<0.01; HFHFr, high-fat, high-fructose diet group; HFHFr-V, HFHFr diet with voglibose group; HFHFr-P, HFHFr diet with pioglitazone group

**Figure S3. Raw images of Western blot with markings for groups and sizes**

**
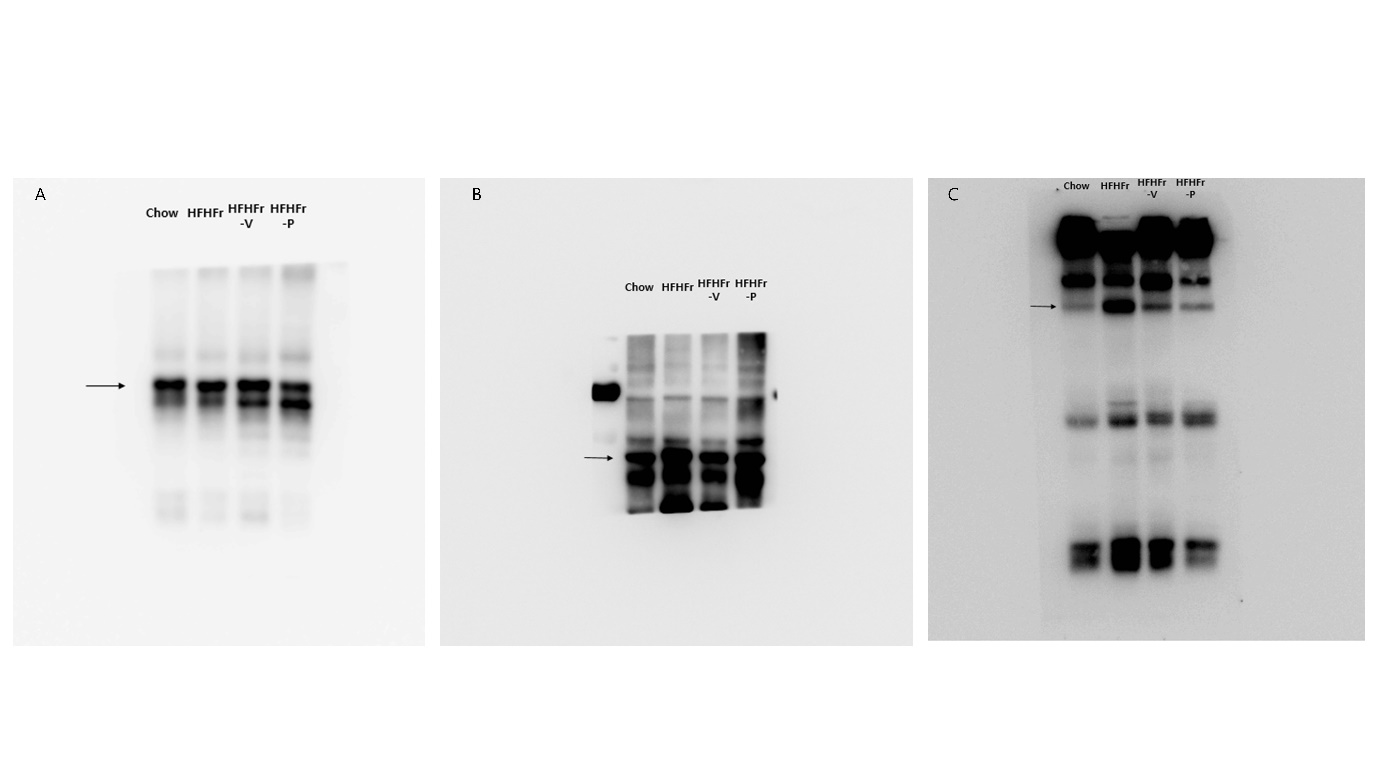
**

Blot images of (A) GAPDH, (B) sterol regulatory element-binding transcription factor-1 (SREBP-1), and (C) carbohydrate response element-binding protein (ChREBP)

HFHFr, high-fat, high-fructose diet group; HFHFr-V, HFHFr diet with voglibose group; HFHFr-P, HFHFr diet with pioglitazone group

**Figure S4. Effects of voglibose on hepatic gluconeogenesis.**


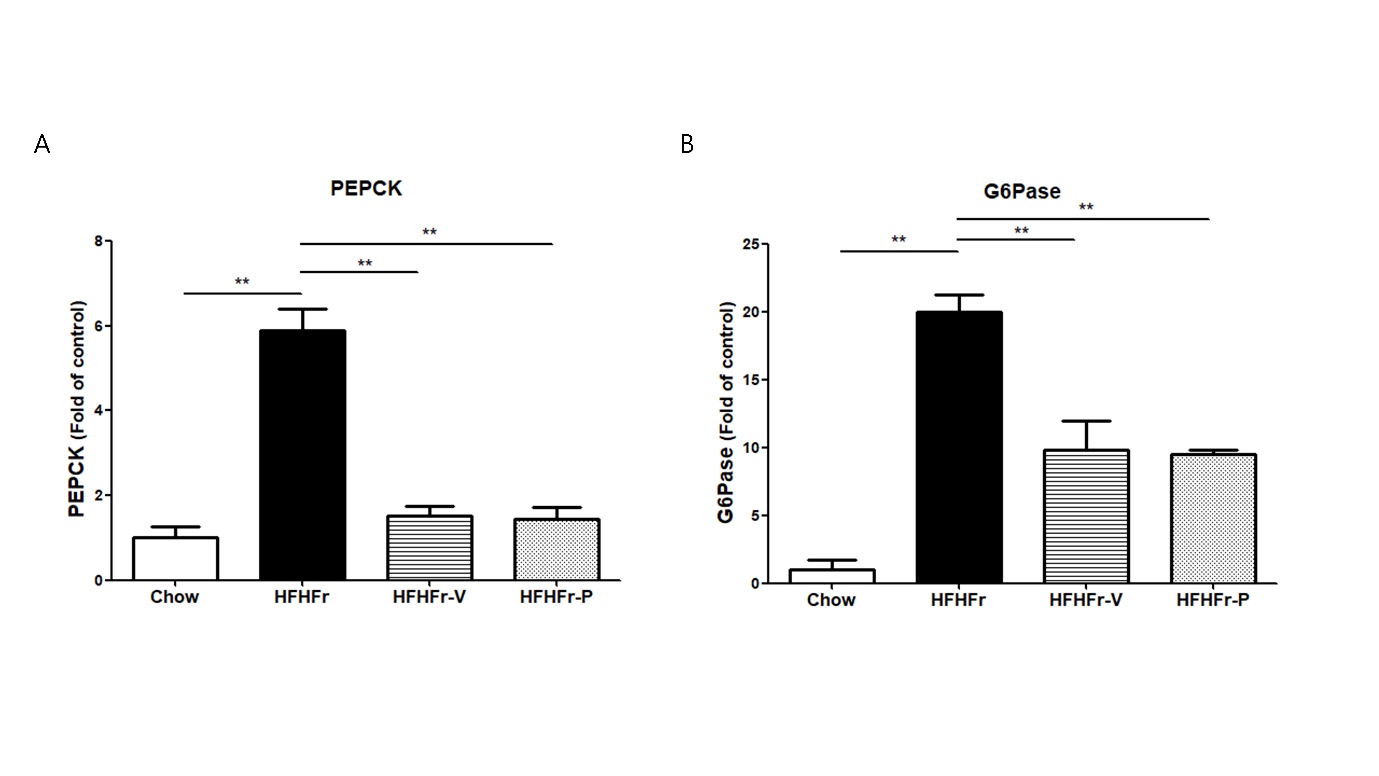


mRNA expression levels of (A) phosphoenolpyruvate carboxykinase (PEPCK) and (B) glucose 6-phosphatase (G6Pase)

*p<0.05; **p<0.01; HFHFr, high-fat, high-fructose diet group; HFHFr-V, HFHFr diet with voglibose group; HFHFr-P, HFHFr diet with pioglitazone group
