# Supplementary material for: The effects of the voglibose on non-alcoholic fatty liver disease in mice model
Source: Sci Rep. 2022 Aug 10;12:13595. doi: 10.1038/s41598-022-15550-7 (PMC9365779; doi:10.1038/s41598-022-15550-7)
Supplement: Supplementary file 2 — Supplementary Information 2. [file 41598_2022_15550_MOESM2_ESM.docx]

**Supplementary Table 1. Primer sequences used in this study**

| Primer |  | Sequence |
| --- | --- | --- |
| 18S | Forward | 5’ -GATGTGAAGGAAGTACAG-3’ |
|  | Reverse | 5’ -CTTCTTGGATACACCCACAGTTC-3’ |
| *Il-1β* | Forward | 5’ -CTGGTGTGTGACGTTCCCATTA -3’ |
|  | Reverse | 5’ -CCGACAGCACGAGGCTTT -3’ |
| *Mcp-1* | Forward | 5’ -ATCCCAATGAGTAGGGTGGAGAGG -3’ |
|  | Reverse | 5’ -CAGAAGTGCTTGAGGTGGTTGTG -3’ |
| *Tgf-β* | Forward | 5’ - AAGAAGTCACCCGCGTGCTA -3’ |
|  | Reverse | 5’ -TGTGTGATGTCTTTGGTTTTGTCA -3’ |
| *α-Sma* | Forward | 5’ -CGTGGCTATTCCTTCGTTAC-3’ |
|  | Reverse | 5’ -TGCCAGCATGACTCCATCC-3’ |
| *Col1a1* | Forward | 5’ -CCTGGTAAAGATGGTGCC -3’ |
|  | Reverse | 5’ -CACCAGGTTCACCTTCGACC -3’ |
| *Srebp-1* | Forward | 5’ -CGCAAGCTGTCGGGGTAG -3’ |
|  | Reverse | 5’ -GTTGTTGATGAGCTGGAGCA -3’ |
| *Chrebp* | Forward | 5’ -CCACAGCGGACACTTCATGG -3’ |
|  | Reverse | 5’ -AGGCTCTCCAGATGGCGTTG -3’ |
| *Acc* | Forward | 5’ -ATGGGCGGAATGGTCTCTTTC -3’ |
|  | Reverse | 5’ -TGGGGACCTTGTCTTCATCAT -3’ |
| *Fas* | Forward | 5’ -GGAGGTGGTGATAGCCGGTAT -3’ |
|  | Reverse | 5’ -TGGGTAATCCATAGAGCCCAG -3’ |
| *PEPCK* | Forward | 5’ -CTGCATAACGGTCTGGACTTC -3’ |
|  | Reverse | 5’ -CAGCAACTGCCCGTACTCC -3’ |
| *G6Pase* | Forward | 5’ -CGACTCGCTATCTCCAAGTGA -3’ |
|  | Reverse | 5’ -GTTGAACCAGTCTCCGACCA -3’ |

18S, 18S ribosomal RNA; Il-1β, interleukin-1 beta; Mcp-1, monocyte chemoattractant protein-1; Tgf-β, transforming growth factor-β; α-Sma, α-smooth muscle actin; Col1a1, collagen type 1α1 chain; Srebp-1, sterol regulatory element-binding transcription factor-1; Chrebp, carbohydrate response element binding protein; Acc, acetyl-CoA carboxylase; Fas, fatty acid synthase; PEPCK, phosphoenolpyruvate carboxykinase; G6Pase, glucose 6‐phosphatase
